# An Update on Molecularly Imprinted Polymer Design through a Computational Approach to Produce Molecular Recognition Material with Enhanced Analytical Performance

**DOI:** 10.3390/molecules26071891

**Published:** 2021-03-26

**Authors:** Shendi Suryana, Mutakin, Yudi Rosandi, Aliya Nur Hasanah

**Affiliations:** 1 Department of Pharmaceutical Analysis and Medicinal Chemistry, Faculty of Pharmacy, Padjadjaran University, Jl. Raya Bandung Sumedang KM 21, Sumedang 45363, Indonesia; shendi@uniga.ac.id (S.S.); mutakin@unpad.ac.id (M.); 2 Pharmacy Department, Faculty of Mathematics and Natural Sciences, Garut University, Jl. Jati No.42B, Tarogong, Garut 44151, Indonesia; 3 Geophysic Department, Faculty of Mathematics and Natural Sciences, Padjadjaran University, Jl. Raya Bandung Sumedang KM 21, Sumedang 45363, Indonesia; rosandi@geophys.unpad.ac.id; 4 Drug Development Study Center, Faculty of Pharmacy, Padjadjaran University, Jl. Raya Bandung Sumedang KM 21, Sumedang 45363, Indonesia

**Keywords:** molecularly imprinted polymer, computational method, DFT

## Abstract

Molecularly imprinted polymer (MIP) computational design is expected to become a routine technique prior to synthesis to produce polymers with high affinity and selectivity towards target molecules. Furthermore, using these simulations reduces the cost of optimizing polymerization composition. There are several computational methods used in MIP fabrication and each requires a comprehensive study in order to select a process with results that are most similar to properties exhibited by polymers synthesized through laboratory experiments. Until now, no review has linked computational strategies with experimental results, which are needed to determine the method that is most appropriate for use in designing MIP with high molecular recognition. This review will present an update of the computational approaches started from 2016 until now on quantum mechanics, molecular mechanics and molecular dynamics that have been widely used. It will also discuss the linear correlation between computational results and the polymer performance tests through laboratory experiments to examine to what extent these methods can be relied upon to obtain polymers with high molecular recognition. Based on the literature search, density functional theory (DFT) with various hybrid functions and basis sets is most often used as a theoretical method to provide a shorter MIP manufacturing process as well as good analytical performance as recognition material.

## 1. Introduction

A molecularly imprinted polymer (MIP) is a synthetic material that has molecular recognition ability with high affinity and selectivity for a particular molecule (template) through the formation of active sites with the shape, size and pattern of functional groups that are complementary to the template used during synthesis [1,2,3]. As an artificial receptor, MIPs have been developed for a variety of applications including chromatography, solid phase extraction, enzyme-linked catalysis, sensor technology, biomimetic sensors, and immunoassays [4]. The MIP components consist of the target template, a functional monomer, crosslinker, polymerization initiator, and porogen [5]. The effectiveness and selectivity of an MIP are greatly influenced by variations in the components [6]. As a polymer sorbent, MIPs are characterized by appropriate sorptive and physicochemical properties, like high binding capacity, large surface area and porosity [7]. The selection of proper components is critical for the synthesis of an MIP with desirable characteristics [8]. The quality of the interactions between the template and functional monomer influences an MIP’s affinity and thus determines its properties, such as the accuracy and selectivity of the recognition sites [9]. Therefore, without a rational MIP design approach, monomer selection often relies on tedious trial and error [10]. Many operations must be conducted and a large number of substrate configurations (in relation to both the type and quantity of individual substrate) must be tested. Experimentally, optimization of monomer composition has centered on combinatorial synthesis and screening [11]. This method relies on a number of different polymers that must be synthesized to obtain the correct monomer. Several studies have shown that MIP development requires up to a dozen different polymers in order to obtain the most suitable composition for optimal performance [12,13,14]. Hence, using computation in this design will ensure that high-affinity polymers with a rational design are produced, an endeavor that saves time and resources [15,16,17,18,19,20]. Using computers as research tools allows performing a large number of calculations and simulations in order to select the most suitable structure and composition of functional monomer, template, crosslinker, and porogen/solvent [21]. The core of such calculations is based on input data related to the chemical composition, the structure at the atomic scale, the distribution of electronic charges leading to dipole formation as well as weak, long range dispersion interactions governed by hydrophobic van der Waals forces [22]. All these factors contribute to a direct picture of a molecular association and the corresponding interaction energy. The interaction energy is calculated as the difference between complex total energy and the reagent energy [23]. On the basis of the strongest interaction (the most negative energy), one selects the most stable complex that is formed by the template and the functional monomer [24]. 

The computational approach to design MIPs generally employs molecular modeling (quantum mechanics), molecular mechanics or molecular dynamics [25]. Quantum mechanics includes ab initio approaches and semiempirical and functional density strategies. Ab initio approaches have been used by several researchers [26,27,28] to determine the ideal monomer and the optimal template to functional monomer ratio. Those researchers have used the Hartree–Fock (HF) method with the 6-31G(d) basis set. The semiempirical approach has been carried out using Austin Model 1 (AM1) and Parameterized Model 3 (PM3). The AM1 approach has been studied by Fu et al. [29], Holdsworth et al. [30] and Baggiani et al. [31], while PM3 has been used by Wang et al. [32]. The most extensively applied technique for MIP design is the density functional theory (DFT) approach, denoted by the numerous MIP publications with optimizations that have used it with various hybrid functions and different basis sets [4,33,34,35]. These studies that have succeeded in producing polymers with high specificity and selectivity to target compounds have shown a correlation between computational and experimental results.

Apart from using quantum mechanics, some researchers have also used molecular mechanics [36,37] and molecular dynamics [38,39,40,41]. Another approach is utilizing a combination of quantum mechanics and molecular dynamics methods to improve the efficiency of the calculation time [42].

The last major review on the computational approach of MIP design was published by Cowen et al. [43]. The focus of that review was an examination of applications of computational methods in MIP design, with an emphasis on theoretical modeling as an alternative to empirical screening. Previous reviews on the use of computational chemistry in imprinted polymer studies have not linked computational results with experimental results, which are needed to determine the method that is most appropriate for use in MIP design. Hence, this review examines recent applications of the computational method in MIP design, with studies from 2016 until now, to determine the major differences in computational approaches since the last major review published by Cowen et al. [43]. This review aims to determine the most efficient computational method in the design of MIPs to achieve a selective material with sensitive analytical performance.

## 2. Quantum Mechanics Methods to Design MIPs

The formation of a complex between a template and a functional monomer is the first step in MIP preparation. Thus, identifying functional monomers that have a strong interaction with the template is crucial for the success of MIP synthesis [44]. The binding energy is used to evaluate quantitatively this interaction [45].

Quantum mechanics is also known as the electronic structure method; it is the most popular MIP design and evaluation technique [46]. In addition, the process describes the electronic structure of a system, so it can better explain noncovalent interactions between a template and a monomer in a pre-polymerized mixture [47]. Molecular modeling of MIP design based on selection of a suitable calculation method is related to the choice of an appropriate basis set [48]. 

Both prior to and after 2016, quantum mechanics methods used to design MIPs have employed ab initio, semiempirical and DFT approaches. The latter is the most frequently used method to design MIPs, in particular to find the best functional monomer and optimal template to functional monomer ratio. A DFT approach provides harmony between accuracy and computational expense. The difference in DFT methods employed before and after 2016 lies in the hybrid functions that are chosen and the basis function. 

### 2.1. Ab Initio Approach to Design MIPs

The ab initio approach has been used to calculate the template–functional monomer complex binding energy to select the best functional monomer with a strong interaction with template or to determine the optimal template to functional monomer ratio. Prior to 2016 and until now, the ab initio approach to design MIPs has used HF/6-31G(d) and HF/3-21G. These approaches have been used mainly to select the best functional monomer based on calculating the monomer–template complex binding energy with various functional monomers; the most negative value indicates the strongest interaction. Besides, the method has also been used to determine the best template to functional monomer ratio, determined from the binding energies considering several ratios. Table 1 presents a summary of the ab initio methods used before and after 2016. Overall, researchers have used HF/3-21G to choose the best functional monomer and HF/6-31G to determine the best template to functional monomer ratio.

#### Examples of the Ab Initio Approach to Design MIPs

The ab initio approach with the HF method with the 3-21G basis set has been used by Hasanah et al. [51,52] and Luo et al. [50]. Another basis set, namely 6-31G(d), has been used by Saad et al. [49] and Hosny et al. [53]. 

Hasanah et al. [51] used the HF approach with the 3-21G basis set for geometry optimization of atenolol (ATE) as a template and itaconic acid (IA) and methacrylic acid (MAA) as functional monomers, while the interaction energy calculations were performed with the Autodock tool. Based on the computational calculations, the ATE–IA complex had a smaller interaction energy (−2.0 kcal/mol) than the ATE–MAA complex (−1.5 kcal/mol). Meanwhile, synthesis of the MIP with the IA monomer showed a recovery of 93.65% ± 1.29% and an imprinting factor (IF) of 11.02; these values were greater than the polymer prepared with MAA: 92.20% ± 1.36% and 1.86, respectively. The IF shows a particular analyte’s distribution ratio on the imprinted polymer as well as under the same conditions as the nonimprinted polymer (NIP) [54]. In another study, Hasanah et al. [52] used the same method to determine the most suitable functional monomer for a MIP that recognizes diazepam by screening acrylamide (AAM) and methyl methacrylate (MMA). From these calculations, the diazepam-MAA complex had a greater interaction energy (−2.0 kcal/mol) than the diazepam-AAM complex (−1.8 kcal/mol). Subsequently, the two polymeric compounds were synthesized and tested for binding properties. The results showed that the adsorption capacity (Q) was greater with MAA (1.1607 mg/g) than AAM (0.1154 mg/g). The simulations were in accordance with the experimental results. 

Furthermore, several studies have optimized the solvent besides the functional monomer in calculations to obtain more accurate prepolymerization simulation results. Luo et al. [50] used HF/3-21G combined with the polarizable continuum model (PCM) to design a MIP to extract acephate residues from contaminated waters. Their goal was to select the best functional monomer to bind with acephate as a template. Their computational approach screened five monomers: acrolein (−4.699 kcal/mol), acrylonitrile (−4.249 kcal/mol), acrylamide (−3.79 kcal/mol), acrylic acid (−7.713 kcal/mol), and MAA (−7.98 kcal/mol) in chloroform as the solvent. According to the principle of choosing a monomer, MAA, with the highest ∆E, is more likely to form strong complexes with the template molecule. Furthermore, the polymer was synthesized with MAA and had an absorption capacity (Q) of 6.59 mg/g and an IF of 2.133. The computational results with the largest interaction energy (∆E) also produced the largest IF values when conducting laboratory experiments. Hence, the computational and practical results were in agreement.

Saad et al. [49] evaluated the functional monomer to template ratio on a MIP for selective extraction of rosmarinic acid using 4-vinyl pyridine (4-VP) and MAA as the functional monomer. The researchers used the HF method with the 6-31G(d) basis set for geometry optimization. Multiple molar ratios were screened, and binding energies of complexes were calculated in the solvent phase using the PCM. A 1:5 ratio for 4-VP had a binding energy of −32.24 kcal/mol in dimethyl sulfoxide (DMSO) and a 1:4 ratio for MAA had a binding energy of 26.74 kcal/mol in DMSO. Subsequently, the computational findings were practically evaluated by comparing the binding performance of the two synthesized polymers with the above-mentioned 4-VP and MAA ratios. The binding test showed that the adsorption capacity (Q) of the 1:5 ratio for 4-VP and the 1:4 ratio for MAA were 34.08 ± 1.54 and 11.48 ± 0.28 mg/g, respectively. This indicates that the simulations were in accordance with the experimental results.

Hosny et al. [53] used the HF approach with the 6-31G(d) basis set combined with the PCM to determine the optimal template to functional monomer molar ratio in design a MIP for extraction of sinapic acid. The computational results revealed the 1:4 sinapic acid (template) to 4-VP molar ratio had the highest binding energy (−145.1 kcal/mol). Subsequently, five polymers were synthesized using a bulk polymerization method. Evaluation of the synthesized polymer binding performance was carried out using a batch rebinding assay, which showed that the 1:4 sinapic acid to 4-VP molar ratio had the highest adsorption capacity (Q) and IF values (2.24 mg/g and 1.006, respectively). These findings underscore that the computational results are relevant to the experimental results. A summary of studies that have used ab initio methods to design MIPs is presented in Table 2.

To determine which ab initio method is best to design MIPs, we must consider the agreement between computational expense, accuracy of calculations and the level of compatibility between theoretical calculations and experimental performance. From the above-mentioned ab initio methods, HF/6-31G(d) presents better accuracy but needs more computational time, while HF/3-21G shows good agreement between the simulation and experimental results and offers a more efficient computational expense.

### 2.2. Semiempirical Approach to Design MIPs

Like the ab initio method, this approach has also been used to calculate binding energy. While the semiempirical approach calculates binding energy faster than the ab initio approach, the accuracy is dependent on the availability of parameters suitable for the molecule being analyzed. If the target molecule is similar to a molecule that exists in the database libraries used in the method parameterization, the result will be good. If the molecules differ significantly from the molecules used in the parameterization method, the answer may be very different from the experimental data [55]. Before and after 2016, the semiempirical approach in MIP design has been generally performed using either AM1 or PM3. Prior to 2016, AM1 was more widely used than PM3, but since 2016 the opposite trend has occurred. Both AM1 and PM3 are based on the modified neglect of differential overlap (MNDO) approach. While AM1 is parameterized largely based on a small number of atomic data, PM3 is parameterized to reproduce a large number of molecular properties. The accuracy of thermochemical predictions with PM3 is slightly better than that of AM1. Until now, these methods have been used to calculate the binding energy of the template–functional monomer complex. A comparison of the semiempirical approach before and after 2016 is shown in Table 3.

#### Examples of the Semiempirical Approach Used to Design MIPs

AM1 has been used to design MIPs by Ishak et al. [56], while PM3 has been used by Li et al. [57], Krishnan et al. [60], Ao et al. [58], Schwarz et al. [59] and Peng et al. [61]. The semiempirical approach has generally been used to calculate the binding energy of prepolymerization complexes. 

Ishak et. al. [56] used AM1 to determine the functional monomer with the strongest interaction with sodium nitrate as the template. The theoretical results showed that allylthiourea had the largest interaction energy towards sodium nitrate (−39.79 kcal/mol) with a 1:4 template to functional monomer molar ratio. Rebinding experiments were carried out to evaluate the binding capacity of the polymer. The experimental binding result showed a Q of 23.75 mg/g and an IF of 1.22. There was good agreement between the calculations and experimental results. 

Li et al. [57] used PM3 to design MIP to extract ginkgolide B. Based on the calculations, the four best functional monomers forming the strongest complex with the template molecule were methacrylamide (−4.6788 kcal/mol), 2-vinylpyridine (−3.7023 kcal/mol), 4-vinyl benzoic acid (−3.5949 kcal/mol), and methyl acrylic acid (−2.7443 kcal/mol). A MIP was synthesized with methacrylamide as the monomer and then subjected to experimental binding. The polymer performed well, with a high adsorption capacity (23.22 mg/g) and an IF of 2.917. Hence, the computational calculations were in agreement with the experiment findings. 

Schwarz et al. [60] used PM3 to aid the selection of the comonomer type as well as to choose the comonomer to template ratios for the formation of the prepolymerization complex to design a MIP that recognizes phytosterol. *N*,*N*^I^-dimethylacrylamide (*N*,*N*^I^-DMAAM) was selected as the functional monomer due to its great binding energy (−8.867 kcal/mol); it formed a stable complex with stigmasteryl methacrylate as a polymerizable template. To confirm the in silico modeling prediction, two MIP preparations were investigated, one with and the other without the functional comonomer *N*,*N*^I^-DMAAM. In addition, three different NIPs were prepared to examine the extent of nonspecific binding that contributes to the overall interaction performance, and in particular the role that the functional and cross-linking monomers play with respect to these interactions. Evaluation of the binding of stigmasterol to each MIP and NIP was conducted via static batch binding. These assays revealed that polymer containing *N*,*N*^I^-DMAAM comonomers exhibited a greater binding capacity (5.6 mg/g) and IF (7.1). This demonstrated the harmony between the computational simulation and the laboratory experiment results. Table 4 shows a summary of studies that have used the semiempirical approach to design MIPs.

The use of the semiempirical approach in MIP design has shown a slight decline in recent years. However, PM3 is still used frequently to calculate the binding energy of template–functional monomer complexes to select the best functional monomer. Both AM1 and PM3 are efficient to use in MIP design because their computational costs are relatively inexpensive. Of note, PM3 is slightly more accurate in calculating binding energy than AM1.

### 2.3. DFT Approach to Design MIPs

The DFT is the most commonly applied theoretical method because it often provides an excellent balance between computational cost and accuracy [62]. Hybrid DFT, especially B3LYP, has frequently been used to calculate binding energy to design highly selective MIPs [35,63,64]. Prior to 2016, B3LYP with various basis sets—for example, B3LYP/6-31G(d), B3LYP/6-31G(d,p) and B3LYP/6-31G+(d,p)—had been widely used in order to select best functional monomer or optimal template to functional monomer ratio [65,66,67,68,69]. However, after 2016, the tendency to use this method has been replaced by other functionals. This is because B3LYP was not properly described with regard to dispersion interaction, a factor that has a significant role in noncovalent interactions [70]. Other hybrid functions such as ωB97xD and M062X represent alternative methods that better describe dispersion interactions [71,72,73,74]. ωB97xD is a long-range corrected hybrid density functional with damped atom–atom dispersion corrections and is more reliable for the calculation of the dispersion than earlier density functionals [75]. M062X is a set of four meta-hybrid generalized gradient approximation (GGA) and meta-GGA DFT functionals [76]. These functionals also yield good results for systems containing dispersion forces [77,78,79,80]. To select the appropriate DFT method, Khan et al. [81] used several methods—B3LYP, BHandHLYP, M062X, and ωB97xD—and basis sets—6-31G(d,p) and 6-31++G(d,p)—to optimize of the template structure. They analyzed and compared the optimized results with earlier experimental data available in the literature. The M062X/6-31G(d,p) method generated a structure with only a very slight deviation from the experimental data and was faster compared with the existing methods. The M062X functional demonstrates better performance in the calculation of relative energies and geometric structures compared to that of other existing functionals. Table 5 shows the use of the DFT approach to design MIP before and after 2016.

#### Examples of the DFT Approach Used to Design MIPs

The DFT method with the ωB97XD hybrid function and the 6-31G(d,p) basis set was employed to simulate binding sites, binding energy, the number of hydrogen bonds, the imprinted molar ratio, and the interaction mechanism to design a MIP for chloramphenicol (CAP) [64]. The researchers used the atoms in molecules theory (AIM) to study the nature of the interaction of the template–functional monomer complex. The theoretical calculations revealed that CAP and AAM formed ordered complexes via hydrogen bonds with a 1:7 CAP and AAM molar ratio using trimethylolpropane trimethylacrylate (TRIM) as the cross-linking agent. The CAP–AAM complex (1:7 molar ratio) had the most stable structure, the most hydrogen bonds and the highest ∆E (−85.68 kcal/mol). The theoretical calculations were validated by a rebinding test of the polymer synthesized from AAM as the monomer with different imprinted molar ratios (CAP:AAM = 1:1 and 1:4–1:8). The rebinding test showed that as the imprinted molar ratio increased, the equilibrium adsorption capacities of CAP-MIPs and NIPs gradually increased. However, their adsorption capacity decreased when the imprinted molar ratio was 1:8, indicating that the imprinted 1:7 molar ratio was optimal, with an adsorption capacity of 7.2 mg/g. These experimental findings were in agreement with the simulation results. Moreover, at an identical imprinted molar ratio, the equilibrium adsorption capacity of CAP-MIPs was obviously larger than that of the NIPs, with an IF of 1.8. This implies that the MIP cavities have a specificity and selectively for CAP. The same hybrid function but with a different basis set, namely def2tzvp, was employed to design a simetryn (SIM) imprinted polymer to determine which functional monomer had the highest interaction energy [66]. 

The DFT method with the M062X hybrid function has been used to design MIP for purifying tylosin, namely to choose the best functional monomer with the highest interaction energy; at the same time, the solvent was evaluated using the PCM [67]. MAA was the chosen functional monomer (−13.99 kcal/mol) and DMSO (ε = 46.82) was selected as the solvent. Furthermore, the static adsorption capacity for tylosin in aqueous solution was estimated to be 106.5 mg/g and had the highest IF of 3.6. In the development of ion imprinted hybrid polymer to adsorb cadmium(II), the functional monomer was selected considering calculations based on M062X and the 6-31G/Lan2DZ base set [68]. The functional M062X demonstrated better performance in the calculation of relative energies and geometric structures compared with other existing functionals [69]. The M062X/6-31+G∗(d,p) was used to develop a MIP compatible with an aqueous environment for solid phase extraction of chenodeoxycholic acid (CDCA) [70], specifically to determine the best functional monomer. M062X and 6-31G(d,p) were used to screen functional monomers in the design of a MIP for tetrachlorodibenzo-*p*-dioxin (TCDD) [63]. The same function was used to optimize the ratio between template molecule and functional monomer to design a MIP for dicyandiamide (DCD) [71]. In all of these studies, there was good agreement between the calculations and experimental results. 

GGA of the BLYP functional was employed to explore the intermolecular interactions and recognition properties of a MIP for deltamethrin (DM) [72]. The researchers also analyzed the molecular electrostatic potentials (MEPs), frontier molecular orbitals (FMOs) and Fukui functions of the template and functional monomer. They found that interaction sites play an important role in the recognition process. The DFT was used to design a MIP for dinoteforan (DNF), namely by testing distinct functional monomers in various solvents [73]. Recoveries using rational and nonrational design (without computational approach) for the MIP were compared. The experimental findings showed recoveries of rational MIP was higher than recoveries of nonrational MIP. 

The DFT combined with PM3 was used to optimize the stoichiometric ratio between template and functional monomer to design a MIP for chlorogenic acid (CGA) [61]. The DFT and the restricted Hartree–Fock (RHF) method were used to design a MIP for pseudoephedrine (PSE). The DFT was also used to determine the best polymerization solvent in conjunction with the PCM. In addition, the basis set superposition error (BSSE) of all calculations was measured using counterpoise (CP) correction [74]. Table 6 summarizes how the DFT approach has been used to accelerate the discovery of functional monomers with the best binding to template molecules, to obtain the appropriate functional monomer to template ratio and to find the best solvent type for synthesis when combined with the PCM.

## 3. Molecular Mechanics Approach to Design MIPs

Molecular mechanics methods are simpler options compared with the quantum mechanical approach. Furthermore, calculations are performed faster and more economically when using multicomponent systems to perform computations [95]. The application of this method is mainly for molecules composed of thousands of atoms, organic particles, oligonucleotides, peptides, saccharides, and compounds in the ground state [96]. 

Prior to 2016, molecular mechanics methods used to design MIPs included the CHARMM and AMBER force fields. Since 2016, the OPLS3 and MMFF94x force fields have become popular. In general, these force fields have been used for energy minimizations. There have been differences in the precision of the results, which depends on the level of accuracy expected. The use of the MMFF94x force field in the molecular operation environment (MOE) to minimize small molecules has produced satisfactory results in the evaluation of stability of hydrogen bonds as well as van der Waals adducts. The OPLS3 force field employs over an order of magnitude more reference data and associated parameter types relative to other commonly used small molecule force fields (e.g., MMFF and CHARMM). As a consequence, OPLS3 achieves a high level of accuracy across performance benchmarks that assess small molecule conformational propensities and solvation. Table 7 shows the development of molecular mechanics in MIP design before and after 2016.

### Examples of Molecular Mechanics Methods Used to Design MIPs

Sullivan et al. [98] investigated the protein–monomer binding interaction that may influence the desired specificity of a MIP for myoglobin using molecular mechanics with the OPLS force field. Five acrylamide-based monomers were considered. Minimization of template (myoglobin) and functional monomers was performed using the OPLS3 force field. The potential binding sites of the protein were predicted using the SiteMap program, followed by prediction of the binding-site-specific interactions of each monomer, studied using Glide docking and post-docking molecular mechanics with generalized Born and surface area continuum solvation (MM-GBSA) binding free energy (BFE) calculations. The simulations revealed that NHMAm was the best monomer to produce a MIP. The experimental results showed that the MIP percentage of protein rebinding was 98.9% ± 0.2%, with an IF of 1.90, while for the NIP it was 51.8% ± 0.4%. This means the simulation of computation was in agreement with the real experiment.

Attallah et al. [99] used the MOE with the MMFF94x force field to estimate the interaction energy between the template 6-mercaptopurine and different monomers in different solvent conditions. The monomers tested in this study were AAM, MAA, MMA, acrylonitrile (AN), and divinylbenzene (DVB). Complexes between the template and the monomers were studied in different solvents including acetone, acetonitrile, chloroform, DMSO, and methanol. MAA provided the most negative binding energies, especially in chloroform, followed by AAM. From all these calculations, it was inferred that the optimal molar ratio is 1:3, where all the binding sites of the template were occupied with monomers making hydrogen bonds. Table 8 summarizes how molecular mechanics have been used to design MIPs. The table highlights that the MMFF94x force field is the best choice of force field to determine noncovalent interactions between polymer constituents.

## 4. Molecular Dynamics Approach to Design MIPs

Molecular dynamics use Newton’s equations of motion to simulate computationally the time evolution of a set of interacting atoms. This is a useful technique to determine conformational energies and noncovalent interactions of the complex in the molecular dynamics simulation [45]. Molecular dynamics simulations are powerful tools: they allow researchers to investigate multicomponent systems that comprise thousands of atoms at a reasonable computation expense [100]. 

Prior to 2016, the molecular dynamics methods applied to design MIPs were the Tripos and COMPASS force fields. After 2016, other force fields have been used, namely the AMBER force field. In molecular dynamics, the force field determines the accuracy of a properly equilibrated molecular simulation to reproduce reality. In MIP design with molecular dynamics, researchers have faced an important choice of which force field is best suited to evaluate the monomer–template functional interaction. In general, molecular dynamics simulations used to design MIPs have been carried out in a canonical ensemble at a constant atom number, volume and temperature (NVT). The Tripos force field has been used in the SYBYL program combine with the LEAPFROG algorithm; this method can simultaneously screen many functional monomers in a virtual library. The COMPASS force field utilizes complex intermolecular interactions, which are quite complex compared with other force fields. Several systematic studies have demonstrated the excellent ability of the AMBER force field to reproduce very high-level data for hydrogen bonds and stacking interactions in the gas phase. Table 9 shows the development of molecular dynamics in the design of MIPs before and after 2016.

### Examples of Molecular Dynamics Methods Used to Design MIPs

Paredes-Ramos et al. [19] used molecular dynamics to determine the best functional monomer and the stoichiometric ratio to design a MIP for catechin. Simulations used the Gromacs 5.0 molecular dynamics software package, with the General AMBER Force Field (GAFF). AutoDock Tools was used to calculate the monomer–porogen affinity of the different available sulfur-based monomers. As functional monomers, Ligands 1–6 (LG1-6) were tested with acetone, methanol and tetrahydrofuran to determine functional monomer–porogen affinity. AutoDock calculations revealed that LG3 has great affinity for catechin, with a binding energy of −56.8 kcal/mol. Polymers with LG2 and LG3 were synthesized. From the binding study, the adsorption capacity of the polymer with LG3 as a functional monomer was 25 mg/g—higher than the NIP (15 mg/g)—with an IF value of 1.67, indicating good imprinting [98].

Madikizela et al. [107] employed molecular dynamics simulations to understand the nature of molecular interactions that occur between 2-vinylpyridine as the functional monomer and ketoprofen as the template. Discover Module of Materials Studio (version 7.0) was used to perform the simulations. In this work, the COMPASS force field was used to calculate the intermolecular interaction energy and bond length between ketoprofen and 2-vinylpyridine. The interactions resulted in molecular dynamics simulations using NVT lasting for 100 ps with a time step of 1 fs. Prior to the execution of molecular dynamics simulations, the Mulliken charges for all the atoms present in ketoprofen and 2-vinylpyridine were assigned. Based on the Mulliken charges, hydrogen bonding could occur where the nitrogen atom (6N) of 2-vinylpyridine will accept the proton (33H) from the carboxylic group of ketoprofen. Molecular dynamics simulations confirmed this observation. The obtained binding energy for the complex that is formed by ketoprofen and 2-vinylpyridine was −11.97 kcal/mol. Therefore, these results confirmed hydrogen bonding between ketoprofen and 2-vinylpyridine. The distribution coefficient of synthesized polymer was 1065 mL/g for the MIP, with an IF of 4.018, and 265 mL/g for the NIP. An IF value more than 1 indicates good imprinting [54]. The same method was used in simulations of the 2-VP–efavirenz interaction to design a MIP for extraction of efavirenz from water [86] and also simulations to determine the best functional monomer that strongly binds with minocycline as a template and to optimize the crosslinker ratio [87]. 

MIP simulations using molecular dynamics have shown more realistic interactions between all MIP components. The use of the appropriate force field and software to execute the molecular dynamics process is fundamental for an accurate simulation. The COMPASS force field has shown good linearity between computational cost and the accuracy of the simulation result. Table 10 summarizes all MIP designs using molecular dynamics.

## 5. Comparison between Different Computational Methods in Design of MIP

Quantum mechanics methods, especially DFT approach, with various hybrid functions and basis sets are the most accurate to calculate and simulate interaction of few molecules [62]. Less computational cost and high accuracy of DFT method have made this method widely used in design of molecularly imprinted polymers [63,64]. However, for systems containing large atoms or molecules, molecular mechanics is more efficient in order to simulate interaction and calculate binding energy of components of systems even less accurate than quantum mechanics [95]. Molecular dynamics method is a more realistic method that can simulate the interaction of all components of polymerization systems, explicitly [100]. Even more realistic, molecular dynamics method needs higher computational cost and time spent compared to quantum mechanics and molecular mechanics. To choose the most appropriate method to design of MIP, consideration of the relationship between computational cost, time spent, and number of molecules involved in the system need to be done to achieve high accuracy of MIP result in real experiments.

## 6. Conclusions

Computational methods, including quantum and molecular mechanics as well as molecular dynamics, have been extensively used by researchers to design MIPs. These methods allow researchers to identify monomers exhibiting the best functions and interactions with templates. Of note, there has been excellent correlation between computations and experimental results associated with MIP analytical performance. Despite the possibility of employing numerous quantum mechanical approaches to achieve the best functional monomer through interaction with template molecules, several drawbacks exist. These include limitations in the MIP system, particularly the difficulty in handling the countless molecules required to obtain a comprehensive description of the prepolymerization system, for example, solvent involvement. Moreover, the duration required by this technique is protracted compared with molecular methods. The DFT quantum mechanical approach using various hybrid functionals and basis sets combined with the PCM is able to describe the interaction between functional monomers and template molecules in the presence of varying solvents, a method similar to the actual prepolymerization conditions.

## Figures and Tables

**Table 1:** Comparison of the ab initio approach to design molecularly imprinted polymers (MIPs) before and after 2016.

HF/6-31G(d)				HF/3-21G			
Before 2016		After 2016		Before 2016		After 2016	
Application	Used By	Application	Used By	Application	Used By	Application	Used By
Determine the optimal T:FM molar ratio	Saad et al. [26]	Determine the optimal T:FM molar ratio	Saad et al. [49]	Calculate the binding energy of the T–FM complex	Luo et al. [50]	Calculate the binding energy of the T–FM complex	Hasanah et al. [51,52]
Calculate the binding energy of the T–FM complex	Tadi and Motghari [27] He et al. [28]	Calculate the binding energy of the T–FM complex Determine the optimal T:FM molar ratio	Hosny et al. [53]				

Abbreviations: FM, functional monomer; T, template.

**Table 2:** Molecularly imprinted polymer (MIP) design using ab initio methods.

Approach	Purpose	Template	Screened Functional Monomers	Screened Solvent/Porogen	Template to Functional Monomer Ratio	Monomer Bond Energy ∆E (kcal/mol)	Experimental Results		Compatibility between Computational and Experimental Results	Reference
							Monomers	Synthesized MIP Analytic Performance		
HF/3-21G	Select the best functional monomer	Atenolol	ITA MAA	n.d.	n.d.	ITA: −2.0 MAA: −1.5	ITA	Q: 4.250 mg/g IF: 11.02 Recovery: 93.6% ± 1.29%	Compatible	[51]
HF/3-21G	Select the best functional monomer	Diazepam	MMA AAM	n.d.	n.d.	MMA: −2 AAM: −1.8	MAA AAM	MMA Q: 1.1607 mg/g IF: 1.7 AAM Q: 0.1154 mg/g IF: 1.25	Compatible	[52]
HF/3-21G PCM	Select the best functional monomer and solvent	Acephate	MAA	Chloroform	n.d.	−7.98	MAA	Q: 6.59 mg/g IF: 2.113	Compatible	[50]
HF/6–31 G(d) PCM	Determine the optimal template to functional monomer molar ratio Select the best solvent	Rosmarinic acid	4-VP	DMSO	1:5 for 4-VP 1:4 for MAA	4-VP: −32.24 MAA: −26.74	4-VP MAA	4-VP Q: 34.08 ± 1.54 mg/g IF: no information MAA Q: 11.48 ± 0.28 mg/g IF: no information	Compatible	[49]
HF/6-31G(d) PCM	Select the best functional monomer and solvent Determine the optimal template to functional monomer molar ratio	Sinapic acid	4-VP	DMSO	1:4 for 4-VP	−145.1	4-VP	Q: 2.24 mg/g IF: 1.006	Compatible	[53]

Abbreviations: 4-VP, 4-vinylpyridine; MAA, methacrylic acid; MMA, methyl methacrylate; AAM, acrylamide; DMSO, dimethyl sulfoxide; Q, binding capacity; IF, imprinting factor; ITA, itaconic acid; HF, Hartree–Fock; PCM, polarizable continuum mode; n.d., not determined.

**Table 3:** Comparison of the semiempirical approach to design molecularly imprinted polymers (MIPs) before and after 2016.

AM1				PM3			
Before 2016		After 2016		Before 2016		After 2016	
Application	Used By	Application	Used By	Application	Used By	Application	Used By
Calculate the binding energy of the T–FMcomplex	Fu et al. [29] Holdsworth et al. [30] Baggiani et al. [31]	Calculate the binding energy of the T–FM complex	Ishak et al. [56]	Calculate the binding energy of the T–FMcomplex	Wang et al. [32]	Calculate the binding energy of the T–FMcomplex	Li et al. [57] Ao et al. [58] Schwarz et al. [59]
						Calculate the binding energy of the T–FMcomplex Determine the optimal T:FM molar ratio	Krishnan et al. [60]
						Determine the optimal T:FM molar ratio	Peng et al. [61]

Abbreviations: AM1, Austin Model 1; PM3, Parameterized Model 3; T, template; FM, functional monomer.

**Table 4:** Molecularly imprinted polymer (MIP) design using semiempirical methods.

Approach	Purpose	Template	Screened Functional Monomer	Template to Functional Monomer Ratio	Screened Monomer Bond Wnergy ∆E (kcal/mol)	Experimental Results		Compatibility between Computational and Experimental Results	Reference
						Types of Functional Monomers	MIP Analytic Performance		
AM1	Select the best functional monomer	Sodium nitrate	Allylthiourea	n.d.	−39.79	Allylthiourea AM; AA; MAA	Q: 23.75 mg/g IF: 1.22	Compatible	[56]
PM3	Select the best functional monomer	Ginkgolide B	MAM; 2-VP; 4-VBA; MAA	n.d.	MAM: −4.6788 2-VP: −3.7023 4-VBA: −3.5949 MAA: −2.7443	MAM	Q: 23.22mg/g IF: 2.917	Compatible	[57]
PM3	Select the best functional monomer Determine the optimal template to functional monomer molar ratio	Andrographolide	MAA	1:3	−24.27	MAA	Q: 0.149 mg/g	Compatible	[60]
PM3	Select the best functional monomer	Domoic acid	TFMAA	n.d.	−7.179	TFMA; MAA; HEMA; AAM; AA	Q: 0.875 mg/g IF: no information	Compatible	[58]
PM3	Select the best functional monomer	Stigmasterol	*N*,*N*^I^-DMAAM	n.d.	−8.867	*N*,*N*^I^-DMAAM MAA	Q: 5.6 mg/g IF: 7.1	Compatible	[59]
PM3	Determine the optimal template to functional monomer molar ratio	Chlorogenic acid	4-VP	1:5	−21.96	4-VP	Q: 42.22 mg/g IF: 2.17	Compatible	[61]

Abbreviations: MAM, methacrylamide; AAM, acrylamide; 2-VP, 2-vinylpyridine; 4-VBA, 4-vinyl benzoic acid; MAA, methacrylic acid; Q, binding capacity; TFMAA, 2-trifluoromethyl acrylic acid; HEMA, hydroxyethyl methacrylate; AA, acrylic acid; *N*,*N*^I^-DMAAM, *N*,*N*^I^-dimethylacrylamide; 4-VP, 4-vinyl pyridine; IF, imprinting factor; n.d., not defined.

**Table 5:** Comparison of the density functional theory (DFT) approach to design molecularly imprinted polymers (MIPs) before and after 2016.

B3LYP/6-31G(d)/B3LYP/6-31G (d,p)/B3LYP/6-31G+(d,p)				B3LYP/6-311G(d,p)/B3LYP/6-311G(d)				ωB97XD/6-31G(d,p)/ωB97XD/def2tzvp				M062X/6-31+G∗(d,P)/M062X/6-31G/Lan2DZ			
Before 2016		After 2016		Before 2016		After 2016		Before 2016		After 2016		Before 2016		After 2016	
Application	Used By	Application	Used By	Application	Used By	Application	Used By	Application	Used By	Application	Used by	Application	Used By	Application	Used By
Calculate the binding energy of the T–FM complex	Pardeshi et al. [4] Dong et al. [34] Gholivand et al. [35]	Calculate the binding energy of the T–FM complex	Li et al. [82] Bujak et al. [83]	-	-	Calculate the binding energy of the T–FM complex Determine the optimal T:F molar ratio	Mehdipour et al. [84] Silva et al. [85] Rahmani et al. [63]	-	-	Calculate the binding energy of the T–FM complex	Tong et al. [86]	-	-	Calculate the binding energy of the T–FM complex	Zeng et al. [78] Adauto et al. [87] Yu et al. [88]
Find a good solvent	Dong et al. [33]	Determine the optimal T: FM molar	Pereira et al. [89] Mehamod et al. [90] Xie et al. [91]	-	-			-	-	Determine the optimal T:FM molar ratio	Liu et al. [72]	-	-	Calculate the binding energy of the T–FM complex Determine the optimal T:FM molar ratio	Khan and Pal [81]
Calculate the binding energy of the T–FM complex Find a good solvent	Barros et al. [92]			-	-			-	-			-	-	Determine the optimal T:FM molar ratio	Liu et al. [93]

Abbreviations: T, template; FM, functional monomer.

**Table 6:** Molecularly imprinted polymer (MIP) design using the density functional theory (DFT) methods.

Approach	Purpose	Template	Screened Functional Monomers	Screened Solvent/Porogen	Template to Functional Monomer Ratio	Screened Monomer Bond Energy ∆E (kcal/mol)	Experimental Results		Compatibility between Computational and Experimental Results	Reference
							Functional Monomers	Synthesized MIP Analytic Performance		
DFT ωB97XD/6-31G(d,p) PCM	Determine the optimal template to functional monomer molar ratio	Chloramphenicol	AAM	ACN	1:7	−85.68	AAM	Q: 7.2 mg/g IF: 1.8	Compatible	[72]
DFT M062X PCM	Select the best functional monomer	Tylosin	MAA	DMSO	n.d.	−13.99	MAA	Q: 106.5 mg/g α: 3.3	Compatible	[78]
DFT ωB97XD/def2tzvp	Select the best functional monomer	Simetryn	MAA	n.d.	1:3	−49.50	MAA	Q: 2.35 mg/g IF: 2.55	Compatible	[86]
DFT M062X and 6-31G/Lan2DZ PCM	Select the best functional monomer	Cadmium	VIN	Ethanol	n.d.	−21.86	VIN	Q: 4.73 mg/g IF: 1.25	Compatible	[87]
M062S/6-31 + g∗(d, P) level	Select the best functional monomer	Chenodeoxycholic acid	DMAEMA	Chloroform	1:5	−14.3369	DMAEMA TFMAA	Q: 49.86 mg/g IF: 2.72	Compatible	[88]
B3LYP/6-31G (d,p) IEFPCM	Determine the optimal template to functional monomer molar ratio	Carvedilol	MAA	Chloroform	1:4	−56.9	MAA	Q: no information IF: no information Kd: 965.10 ml/g	Compatible	[89]
B3LYP/6-31G+(d,p)	Determine the optimal template to functional monomer molar ratio	Deltamethrin	AAM	Chloroform	1:6	n.d.	AAM	Q: 75.72 mg/g IF: no information	Compatible	[91]
B3LYP/6-311G(d) PCM	Select the best functional monomer Determine the optimal template to functional monomer molar ratio	Diazinon	MAA	Chloroform	1:5	−6.739	MAA	Q: no information IF: no information Kd: 2.154 ml/g	Compatible	[84]
B3LYP/6-31 g (d,p)	Select the best functional monomer	*N*-Nitrosodiphenylamine	MAA	n.d.	n.d.	−10.91	MAA	Q: no information IF: no information Recovery: 95% ± 4.5%	Compatible	[82]
B3LYP, BHandHLYP, M062X and ωB97xD methods; 6-31G(d,p) and 6-31++G(d,p) basis sets PCM	Select the best functional Determine the optimal template to functional monomer molar ratio	Dioxin	AAM	ACN	1:4	−25.918	AAM MAA IA VP	Q: 3.7 mg/g IF: 2.371	Compatible	[81]
B3LYP/6-311G(d,p) PCM	Select the best functional monomer Determine the optimal template to functional monomer molar ratio	Dinotefuran	MAA	Chloroform	1:4	−42.4	MAA	Q: no information IF: no information Recovery: 89.87±4.64%	Compatible	[85]
PM3 DFT	Determine the optimal template to functional monomer molar ratio	Chlorogenic acid	4-VP		1:5	−40.16	4-VP	Q: 42.22 mg/g IF: no Information	Compatible	[61]
B3LYP/6-31G (d,p)	Determine the optimal template to functional monomer molar ratio	Pyrogallol	MMA	n.d.	1:3	−24.072	MMA	Q: no information IF: 1.4	Compatible	[90]
RHF DFT/6-31+G** PCM	Select the best functional monomer	Pseudoephedrine	MAA	Methanol	1:2	−12.45	MAA	Q: no information IF: 3.64	Compatible	[94]
B3LYP/6-311G(d) NBO PCM	Select the best functional monomer Determine the optimal template to functional monomer molar ratio	Naltrexone	MAA	THF	1:5	−10.43	MAA	Q: 11.60 mg/g IF: 2.27	Compatible	[63]
M062X/6-31G(d,p) BSSE MEP PCM	Determine the optimal template to functional monomer molar ratio	Dicyandiamide	MAA	ACN	1:5	−45.74	MAA	Q: 17.24 mg/g IF: no information	Compatible	[93]
B3LYP/6-31G (d, p)	Select the best functional monomer	Atropine	TFMAA	n.d.	1:4	−49.62	TFMAA MAA	Q: 47 mg/g IF: 1.6	Compatible	[48]

Abbreviations: B3LYP, Becke 3 lee Yan Par; AAM, acrylamide; THF, tetrahydrofuran; ACN, acetonitrile; α, selectivity coefficient; Q, adsorption capacity; IF, imprinting factor; VIN, 1-vinylimidazole; DMAEMA, 2-(dimethylamino)ethyl methacrylate; AA, acrylic acid; IEFPCM, the integral equation formalism polarizable continuum model; Kd, distribution coefficient; HIMA, 2-hydroxy-3-(isopropylamino)propyl methacrylate; MMA, methyl methacrylate; THF, tetrahydrofuran; NBO, natural bond orbital, BSSE, basis set superposition error; MEP, molecular electrostatic potential; n.d., not defined.

**Table 7:** Comparison of the molecular mechanics approach to design molecularly imprinted polymers (MIPs) before and after 2016.

CHARMM Force Field				AMBER MM Force Field				OPLS3 Force Field				MMFF94x Force Field			
Before 2016		After 2016		Before 2016		After 2016		Before 2016		After 2016		Before 2016		After 2016	
Application	Used By	Application	Used By	Application	Used By	Application	Used By	Application	Used By	Application	Used By	Application	Used By	Application	Used By
Select the best functional monomer	Sobiech et al. [97]	-	-	Selection of the best functional monomer	Farrington et al. [36]	-	-	-	-	Select the best functional monomer	Sullivan et al. [98]	-	-	Select the best functional monomer	Attallah et al. [99]

**Table 8:** Molecularly imprinter polymer (MIP) design using molecular mechanics.

Approach	Purpose	Template	Screened Functional Monomer	Screened Solvent/Porogen	Template to Functional Monomer	Screened Monomer Bond Energy ∆E (kcal/mol)	Experimental Results		Compatibility between Computational and Experimental Results	Reference
							Functional Monomers	Synthesized MIP Analytic Performance		
OPLS3 force field Docking	Select the best functional monomer	Myoglobin	NHEAM	n.d.	1:1	−11.3	AAM; NHMAA; NHEAM; DMAM; TrisNHMA; M; MBAM	IF: 1.3	Compatible	[82]
MMFF94x force field HF/6-31G(d) PCM	Select the best functional monomer	6-mercaptopurine	MAA	Chloroform	1:3		MAA	Q: 0.822 mg/g IF: 3.99	Compatible	[83]

Abbreviations: AAM, acrylamide; NHMAAM, *N*-(hydroxymethyl)acrylamide; NHEAM, *N*-(hydroxyethyl)acrylamide; DMAM, *N*,*N*-dimethylacrylamide; TrisNHMAM, *N*-[Tris(hydroxymethyl)methyl]acrylamide); MBAM, *N*,*N^I^*-methylenebis(acrylamide); MAA, methacrylate acid; PCM, polarizable continuum model; n.d., not defined.

**Table 9:** Comparison of the molecular dynamics approach to design molecularly imprinted polymers (MIPs) before and after 2016.

Tripos Force Field				COMPASS Force Field				AMBER Force Field			
Before 2016		After 2016		Before 2016		After 2016		Before 2016		After 2016	
Application	Used By	Application	Used By	Application	Used By	Application	Used By	Application	Used By	Application	Used By
Select the best FM	Bakas et al. [39]	Select the best FM Determine the optimal T:FM molar ratio	Rodríguez-Dorado et al. [101]	Determine the optimal T:FM:CL ratio	Kong et al. [41]	Investigate natural interactions between T and FM	Madikizela et al. [102]	-	-	Select the best FM	Paredes-Ramos et al. [103]
Select the best FM	Piletska et al. [40]	Select the best FM Determine the optimal T:FM ratio	Viveiros et al. [104]	-	-	Investigate nature interactions between T and FM	Mahlambi et al. [105]	-	-	-	-
						Select the best FM	Eroglu et al. [106]				

Abbreviations: T, template; FM, functional monomer; CL, crosslinker.

**Table 10:** Molecularly imprinted polymer (MIP) design using molecular dynamics.

Approach	Purpose	Template	Screened Functional Monomers	Screened Solvent/Porogen	Template to Functional Monomer Ratio	Screened Monomer Bond Energy ∆E (kcal/mol)	Experimental Results		Compatibility between Computational and Experimental Results	Reference
							Functional Monomers	Synthesized MIP Analytic performance		
AMBER force field	Select the best functional	Catechin	LG1	Methanol	n.d.	−59	LG1	Q: 15 mg/g	Compatible	[19]
Materials Studio with the COMPASS force field	Investigate the nature of interactions between template and functional monomer	Ketoprofen	2-VP	n.d.	n.d.	−11.97	2-VP	Q: no Information Kd: 1065 mL/g IF: 4.018	Compatible	[107]
Discover module of Materials Studio with the COMPASS force field	Investigate the nature of the interaction between template and functional monomer	Efavirenz	2-VP	n.d.	n.d.	−18	2-VP	97% recovery	Compatible	[105]
Materials Studio with the COMPASS force field	Selection of the best functional	Minocycline	AA	n.d.	n.d.	n.d.	AA	IF: 2.4	Compatible	[106]

Abbreviations: NMBAM, *N*,*N*′-methylenebisacrylamide; LG, ligand; 2-VP, 2-vinylpyridine; AA, acrylic acid; Q, binding capacity; Kd, distribution coefficient; IF, imprinting factor; n.d., not defined.

## Data Availability

Data sharing not applicable.
